# Review on Major Food-Borne Zoonotic Bacterial Pathogens

**DOI:** 10.1155/2020/4674235

**Published:** 2020-06-29

**Authors:** Engidaw Abebe, Getachew Gugsa, Meselu Ahmed

**Affiliations:** School of Veterinary Medicine, Wollo University, Dessie, Ethiopia

## Abstract

Food-borne microorganisms are major pathogens affecting food safety and cause human illness worldwide as a result of consumption of foodstuff, mainly animal products contaminated with vegetative pathogens or their toxins. Most of these microbes have zoonotic importance resulting in significant impact on both public health and economic sectors. Bacteria are the causative agents of two-thirds of human food-borne diseases worldwide with high burden in developing countries. Hence, the objectives of this review paper are to highlight the background of food-borne bacterial pathogens and to review common major food-borne zoonotic bacterial pathogens. Food animals are the major reservoirs of many food-borne zoonotic bacterial pathogens, and food products of animal origin are the main vehicles of transmission. Meat, dairy products, and eggs are the main ways by which people are exposed to zoonotic bacteria. *S. aureus*, *Salmonella* species, *Campylobacter* species, *L. monocytogenes*, and *E. coli* are the major zoonotic bacterial pathogens which are the causative agents of food-borne illness and death in the world associated with consumption of contaminated animal products. Production of toxins and structural virulent factors are responsible for the pathogenesis of these bacteria. These major zoonotic bacteria cause human infections which are characterized mainly by gastrointestinal symptoms including nausea, vomiting, diarrhea, abdominal cramps, and other agent-specific symptoms. Some bacteria may cause severe complications. Conventional (culturing), serological, and molecular techniques are important for detection of these common zoonotic bacteria and their toxins in food. Good hygiene, GMP, sanitation in operating procedures, and implementation of standardized HACCP and pasteurization procedures are effective methods for the control and prevention. Currently, the emergence of multidrug-resistant zoonotic bacteria associated with consumption of contaminated animal products is a great concern for the public health, and there should be coordinated surveillance and monitoring system for food-borne zoonotic bacterial pathogens particularly in developing countries including Ethiopia.

## 1. Introduction

Ethiopia is believed to have the largest livestock population in Africa, with an estimated population of 60,392,019 cattle, 31,302,257 sheep, 32,738,385 goats, 2,007,829 horses, 461,665 mules, 8,845,589 donkeys, 1,418,457 camels, 56,056,778 poultry, and 6,523,969 beehives [1]. The livestock sector contributes about 45% to the agricultural gross domestic product (GDP), 18.7% to the national GDP, and 16–19% to the total foreign exchange earnings of the country. It is the source of industrial raw materials (milk, meat, and hides and skin) and high-value protein to potential consumers in Ethiopia [2].

The consumption of animal products like meat, milk, and egg is increased due to rapid human population growth, urbanization, per capita income raise, globalization, and the changes on consumer habits (preference of high-protein diet). This situation results in a high demand of food of animal origin and leads to intensive animal production and processing of products, especially mass production and movement of products globally. During this time, there may be defective processing practices at any point of the farm to fork chain which increase the chances of contamination and spread of food-borne pathogens [3, 4].

Food products may become contaminated at different stages along the food chain [5], could be during production, processing, distribution, preparation, and/or final consumption. The risk of food getting contaminated depends largely on the health status of the food handlers, their personal hygiene, knowledge, and practice of food hygiene [6].

According to World Health Organization (WHO), food-borne diseases are defined as diseases of infectious or toxic nature which are caused by the consumption of food or water [7]. Intoxication (toxin produced by the pathogens causes food poisoning), infection (ingestion of food containing pathogens), and toxicoinfections (producing toxins while growing in the human intestines) are the three types of food-borne diseases [3, 8].

Diseases of animal origin can be transmitted between humans and animals through direct contact, indirect environmental contact, and/or through food consumption [9]. Around 60% of human diseases are originated from animals, and approximately 75% of new emerging human infectious diseases are transmitted from vertebrate animals to humans [10].

Food-borne pathogens are microorganisms (i.e., bacteria, viruses, and fungi) as well as a number of parasites [11], and they are the primary cause of food spoilage and food-borne diseases [5]. Food-borne microbes are major problems affecting food safety and cause human infections after consumption of the animal products contaminated with microorganisms or their toxins [4].

Most of the pathogens have a zoonotic origin, and food products of animal origin are considered as major vehicles of food-borne infections [12]. Food-producing animals (cattle, chickens, pigs, turkeys, etc.) are the major reservoirs for many food-borne pathogens [4]. Animal products (meat, milk, egg, fish, etc.) and their products have high risk due to pathogen contents, natural toxins, adulterants, and other possible contaminants [13], and the risk of food-borne diseases in humans is increasing when consumption of food of animal origin is increased [14].

In recent years, food-borne pathogens become an important public health problem worldwide, and their impact on health (significant morbidity and mortality rate) and economy is increasingly recognized [5, 11, 15–17]. According to different reports, a huge number of people suffer from food-borne diseases each year worldwide [12], and around 600 million (10 people in the world) become ill due to the consumption of contaminated food [18]. Due to unrecognized or unreported outbreaks, statistical data of food-borne diseases are increased [5].

Food-borne diseases are major health problems both in developed and developing countries [19], but developing countries tend to suffer from the largest share of the burden of food-borne diseases [16]. According to the WHO, 30% of the population suffer from food-borne diseases each year in developed countries, and up to 2 million deaths are estimated per year in developing countries [19].

Nowadays, the awareness has been growing on the public health impact of zoonotic food-borne pathogens transmitted from animal-originated food [20]. There is emergence of new pathogens, and the way of transmission of known food-borne pathogens is changing or is now associated with new food vehicles [15]. The prevalence of multidrug-resistant (MDR) food-borne pathogens is increased after consumption of contaminated food due to the use of drugs for human therapy and animal farming which are responsible for more serious disease than susceptible bacteria [5, 21]. Drug resistance among the pathogens in common and food-borne pathogens in particular is an emerging problem [15, 22].

Prevailing poor food handling and sanitation practices, inadequate food safety laws, weak regulatory systems, lack of financial resources to invest in safer equipment, and lack of education for food handlers are the reasons for common occurrence of food-borne diseases in developing countries including Ethiopia [13, 16, 23, 24]. The habit of raw beef consumption [25], overcrowding, poverty, inadequate sanitary conditions, and poor general hygiene are also the factors of food-borne diseases in Ethiopia [26].

The public health importance of several bacterial pathogens associated with food of animal origin has been shown by studies conducted in different parts of the country [13]. However, there is a lack of reliable statistics on food-borne diseases as well as well-organized and documented information on the occurrence of such diseases due to poor or nonexistent reporting systems in Ethiopia as well as in most developing countries.

Therefore, the objectives of this review paper are as follows:

  To highlight about the background information of food-borne bacterial pathogens
  To review common major food-borne zoonotic bacterial pathogens

## 2. Food-Borne Zoonotic Bacterial Pathogens

### 2.1. General Background

Food-borne diseases occur as a result of consumption of contaminated food stuffs especially from animal products [26–29]. Food poisoning syndrome results from ingestion of a wide variety of food contaminated with pathogenic organisms (bacteria, viruses, parasites, and fungi) and their toxin and chemicals. Bacteria (66%), chemicals (26%), virus (4%), and parasites (4%) are the main causes of food-borne diseases [8]. Currently, food-borne disease caused by bacterial contamination is one of the biggest issues affecting human health and food safety [30].

Bacteria are the causative agent of two-thirds of food-borne disease outbreaks though there have been around 250 different food-borne diseases [31]. From the biological hazards, bacterial pathogens are the most serious concern regarding the issues of meat safety to consumers [32]. Bacterial food-borne illnesses are among the most widely spread global public health problems in recent times [8].

Vertebrate animal species are natural reservoirs for many pathogens that cause human infections after transmitted through food [33]. Food of animal origin particularly meat (beef, mutton, and pork), dairy products (milk, cheese, yoghurt, and ice cream), and eggs are the three ways by which people are exposed to pathogenic bacteria [19]. Due to their nutritional value, mainly high protein and lipid content, dairy products are a suitable growth environment for a range of microorganisms [34]. Contaminated raw meat is one of the main sources of food-borne disease [26].

Food of animal origin (milk, meat, and their products) can become contaminated with bacteria during food processing or slaughtering [35]. These pathogens come into contact with food during harvest or slaughtering, processing, storage, and packaging. Environmental challenges have caused food-borne bacterial pathogens to evolve and the susceptibility of the human population to infections [5]. The battle against bacterial food-borne diseases is facing new challenges due to rapidly changing patterns of human consumption, the globalization of the food market, and climate change [31].

Food-borne bacterial diseases caused by bacteria are most commonly prevented and controlled by proper cooking and preparing of food as well as storing. The control method or measures also include education of those who prepare the food at home and other food handlers, prohibiting individuals with abscess or other skin lesions from handling food, and placing of food in a cold place at 4°C or lower temperature which prevent bacterial multiplication and toxin formation. Food must be kept at room temperature for as little time as possible [8].

### 2.2. Common Major Food-Borne Zoonotic Bacterial Pathogens

Among the bacteria that cause food-borne poisoning, some are particularly important in terms of frequency and/or of seriousness of the disease. Miscellaneous bacteria (including Gram positive and Gram negative) produce toxins that cause food-borne poisoning, resulting symptoms ranging from gastrointestinal disorders to paralysis and death [36]. It has been reported that Gram-negative bacteria account for approximately 69% of the cases of bacterial food-borne disease [24].

Although there are 31 pathogens that have been identified as causing food-borne diseases [11], bacterial pathogens including *Staphylococcus aureus* (*S. aureus*), *Salmonella* species, *Campylobacter* species, *Listeria monocytogenes* (*L. monocytogenes*), and *Escherichia coli* (*E. coli*) are the common causes of food-borne diseases and death in the world [5, 11, 20, 27, 37].

#### 2.2.1. *Staphylococcus aureus*

The name *Staphylococcus* (staphyle = bunch of grapes in Greece) was introduced in 1883 by Ogston [31]. *S. aureus* is one of the most common food-borne pathogens worldwide [21, 38] with high occurrence second to salmonellosis [39].

It is a microorganism that is present as a commensal on the skin, nose, and mucous membranes of healthy humans and animals [39, 40]. However, it is a well-known opportunistic food-borne pathogen [41, 42] that can cause multiple infectious diseases of diverse severity [40]. It causes wide spectrum of diseases in both humans and animals [43].

The presence of *S. aureus* in products for human consumption is important to the food industry, as some strains are the cause of food-borne intoxication [21]. They are responsible for food spoilage, reduction of food safety, and shelf life and cause food-borne poisoning [44]. *S. aureus* is a leading cause of food poisoning resulting from the consumption of contaminated food with staphylococcal enterotoxins [45].

It earns public attention due to increasing mortality associated with multidrug resistance [21]. The widespread use of antibiotics and ability of the bacteria to rapidly develop and acquire antimicrobial resistance have facilitated the emergence of resistant strains such as methicillin-resistant *S. aureus* (MRSA) [41, 42, 46]. The emergence of MRSA in livestock and the possibility of human cross-contamination have caused a serious concern [38].

MRSA were first reported in the early 1960s and are now regarded as a major hospital-acquired pathogen worldwide [47]. MRSA is a well-known pathogen occurring both in human and veterinary medicine [21, 48].

*(1) Etiology*. The genus *Staphylococcus* comprises several species and subspecies [44]. *S. aureus* is a Gram-positive, catalase-positive, coagulase-positive, usually oxidase-negative, and facultative anaerobic coccus, which belongs to the family of Micrococcaceae [43, 49]. It is a nonmotile bacterium. Cells are spherical single and often form grape-like clusters [31]. Gold colony pigmentation, production of coagulase, fermentation of mannitol and trehalose, and production of heat stable thermonuclease distinguish *S. aureus* from other staphylococcal species [49]. The organisms are able to grow in a wide range of temperatures (7°C to 48°C with an optimum of 30°C to 37°C), pH (4.2 to 9.3, with an optimum of 7.0 to 7.5), and sodium chloride concentrations (up to 15% NaCl). These characteristics enable the bacteria to survive in a wide variety of food, especially those that require manipulation during processing, including fermented food products like cheese [31].

*(2) Epidemiology*. The epidemiology of this microorganism in animals has gained interest because of its importance in veterinary medicine, the increment of infectious processes caused by this pathogen (especially MRSA strains), and the emergence of some clonal lineages associated with animals, and zoonotic potential evidence is increased in the last years [40]. *S. aureus* is among the leading causes of food-borne bacterial intoxications worldwide [50]. It is one of the most common causes of reported food-borne diseases in the United States [7].

Around 50% of healthy individuals harbor the bacteria in their nasal passage, throat, and skin, whereas the mastitic cow is a common source of *S. aureus* in raw milk [51]. It is widely present in a broad host range, including human beings and food-producing animals, such as pigs, cows, goats, chickens, and ducks [42]. Food contamination with *S. aureus* may occur directly from infected food-producing animals or may result from poor hygiene during production processes or the retail and storage of food [52].

A multifactorial range of independent risk factors for MRSA has been reported in the literature, and factors include immunosuppression hemodialysis, peripheral malperfusion, advanced age, extended in hospital stays, residency in long-term care facilities, inadequacy of antimicrobial therapy, indwelling devices, insulin-requiring diabetes, and decubitus ulcers, among others [53].

Studies that have been conducted in different regions of Ethiopia indicated the occurrence of *S. aureus* in food of animal origin as few of them are given in Table 1.

*(3) Transmission*. *S. aureus* is transmitted from the contaminated animal-source foodstuff [7, 31, 54]. This bacterium has the potential to contaminate animal products and may enter the food chain during processing, preparation, wrapping, mincing, and storage [42].

*S. aureus* food contamination can occur in different kinds of food resources such as livestock and poultry products and seafood, as well as bakery products, which often contain enterotoxigenic strains [38]. Various food items can be contaminated by staphylococcal enterotoxins, especially moist food containing starch and protein [30].

Several food materials including milk and dairy products [19], pork, beef, mutton, poultry, and eggs are common vehicles that are frequently implicated in Staphylococcal food poisoning [42]. Raw meat is a good medium for *S. aureus* survival and spread of drug-resistant *S. aureus* in the community [61]. Additionally, food handlers carrying *S. aureus* on their bodies or gloves can also contaminate food [42].

*(4) Pathogenesis*. *S. aureus* has a variety of virulence factors that, singly and in combination, can result in severe infection [51]. A wide spectrum of secreted and cell surface-associated virulence factors can be expressed to promote adhesion to the host extracellular matrix components, damage host cells, and fight the immune system [62]. The extracellular active substances that the organism produces which are thought to contribute to the pathogenicity of the organism include coagulase, hemolysins, nuclease, acid phosphatase, lipase, protease, fibrinolysin, enterotoxins, and toxic shock syndrome toxins [21].

If food is stored for some time in room temperature, the organism in the food can produce its toxin. The bacteria produce enterotoxin while multiplying in food [31]. The enterotoxins are proteolytic, enzyme-resistant, and heat-stable, and they may be present in food when *S. aureus* are absent [31, 54].

The enterotoxins are classified into 23 different staphylococcal enterotoxins (SEs) and SE-like toxins [30, 41, 42] including the six major serologically different types (A, B, C1, C2, D, and E) that differ in toxicity [31]. However, little is known about the prevalence of MRSA in food involved in international trade or about their enterotoxin production [41].

Enterotoxins stimulate the central nervous system (CNS) vomiting center and inhibit water and sodium absorption in the small intestine [8]. They are responsible for symptoms of acute gastroenteritis [31].

*(5) Symptoms*. The bacteria can cause various diseases with different symptoms, ranging from simple skin infection to more serious and potentially life-threatening infections such as septicemia, necrotizing fasciitis, infective endocarditis, necrotizing pneumonia, and toxic shock syndrome [42, 46].

The disease has short incubation period (clinical signs appear within 2–4 hrs after consumption of food) [8]. It is characterized by nausea and vomiting, mainly subnormal temperature, chills, headache [3], and abdominal cramping with or without diarrhea [7] but no fever [42]. Abdominal cramps, nausea, and vomiting are the most common [7]. Death occasionally occurs among more susceptible individuals, such as children and the elderly population [42].

MRSA strains or multidrug-resistant *S. aureus* cause nosocomial infections responsible for rapidly progressive, potential fatal diseases including life-threatening pneumonia, necrotizing fasciitis, endocarditis, osteomyelitis, severe sepsis, and toxinoses such as toxic shock syndrome [53].

*(6) Detection*. A range of selective and diagnostic media have been developed to assist in the detection and enumeration of *Staphylococci* in routine food surveillance programmes and food poisoning investigations [31]. Diagnostic microbiology laboratories and reference laboratories are the key for identifying outbreaks of MRSA [47].

The most widely used and generally accepted criterion for identification of pathogenic *Staphylococci* is usually by their ability to produce coagulase. A common medium used for the isolation of pathogenic *Staphylococci* is the mannitol salt agar [31]. Currently available standard method for the detection of coagulase-positive *Staphylococci* including *S. aureus* in food is based on selective enrichment and subsequent isolation of colonies with characteristic morphology and identification by microbiological and biochemical-based confirmations [63].

Molecular biological-based and immunological techniques are also considered as important tools for investigating *S. aureus* contaminations [42]. Real-time polymerase chain reaction (RT-PCR) and quantitative PCR are increasingly being employed in clinical laboratories for the rapid detection and identification of MRSA strains. Another common laboratory test is a rapid latex agglutination test [47].

The most important methods used to detect enterotoxins in food are the enzyme-linked immunosorbent assay (ELISA) method and other screening methods [31]. Other serologic tests including agglutination test [30] and gel diffusion can be employed to detect and identify enterotoxins [8]. Molecular biological methods like nucleic acid hybridization, PCR, and fluorescent immunoassays are the most popular approaches for the detection of enterotoxins in recent times [30].

*(7) Prevention and Control*. *Staphylococci* are ubiquitous and are impossible to eliminate from the environment. Prevention of staphylococcal infections/intoxication requires strategies to interrupt various modes of transmission [31]. Cooking food thoroughly, preventing contamination and cross-contamination, and maintaining critical points are effective ways to prevent staphylococcal infections. Public awareness regarding safe meat-handling and other public health interventions could be a cornerstone in preventing the outbreak [7].

Control programs include improvements in personal hygiene practices among healthcare workers and food handlers, decontamination of equipment, surfaces, clothing, judicious use of antibiotics, proper cooking and storage of food, and screening programs [31].

Microbiological guidelines such as Hazard Analysis and Critical Control Points (HACCP), Good Manufacturing Practice (GMP), and good hygienic practices developed by the WHO and the United States Food and Drug Administration should be implemented strictly to prevent *S. aureus* contamination [7]. Areas where MRSA patients are nursed should be thoroughly cleaned using disinfectants [47].

#### 2.2.2. Nontyphoidal *Salmonella*

Nontyphoidal *Salmonella* are most important zoonotic bacterial food-borne pathogens of humans. *Salmonellae* are widely distributed in nature [64], and they are the major pathogenic bacteria in humans as well as in animals [65]. They are most frequently isolated bacterial agents of food-borne disease outbreaks [66], and they account around 93.8 million food-borne illnesses and 155,000 deaths per year worldwide [4]. *Salmonella* has been found to be the major cause of food-borne diseases and a serious public health problem in the world, with an increasing concern for the emergence and spread of antimicrobial-resistant strains [12] including in industrialized countries [67]. Antibiotic-resistant *Salmonella* infections of both humans and animals are universal concerns, particularly in developing countries [12].

Apart from the morbidity and mortality costs in humans and animals, restrictions to trade and discard contaminated food are important socioeconomic problems of the bacteria [68].

*(1) Etiology*. *Salmonella* comprises greater than 2,500 identified serotypes [69] which are included under two species *Salmonella enterica* (*S*. *enterica*) and *Salmonella bongori* (*S. bongori*). *S. enterica* is further classed into six subspecies [3] and is a major pathogen in humans as well as in animals [67, 69]. More than 150 serotypes can cause food-borne salmonellosis [3]. However, *S. typhimurium* and *S. enteritidis* are more common [3, 67, 68]. Those bacteria are Gram-negative, relatively anaerobic, nonspore forming, and straight rods belonging to the Enterobateriaceae family [9] that are indistinguishable from *E. coli* under the microscope or ordinary nutrient media [8].

*(2) Epidemiology*. Salmonella is one of the major public health concerns all over the world [70]. It is the most common food-borne diseases in both developing and developed countries, although incidence rates vary according to the country [65].

The prominent epidemiological factor is the common carrier status in animals [71]. *Salmonella* is found naturally in the environment and in both domestic and wild animals [4]. The primary habitat of *Salmonella* species is the intestinal tract of the animals such as farm animals, humans, birds, reptiles, and insects. Animals are the reservoir of food-borne diseases of *Salmonella* [8].

Nontyphoidal *Salmonella* species are zoonotic agents, and food products of animal origin are the main sources for their transmission [67]. Poultry [66], pigs and cattle [72], and their products like meat, eggs [4, 73], and milk [74] are most commonly identified as food sources responsible for outbreaks of human salmonellosis although the microorganism has also been found in other foodstuff [4]. Chicken products including eggs are widely acknowledged to be a significant reservoir for *Salmonella* and have been consistently implicated in sporadic cases and the outbreak of human salmonellosis [64].

Consumption of raw or unsafe food, cross-contamination, improper food storage, poor personal hygiene practices, inadequate cooling and reheating of food items, and a prolonged time lapse between preparing and consuming food items were mentioned as contributing factors to an outbreak of salmonellosis in humans [12].

The bacteria enter the food chain at any point in livestock feed, and in food manufacturing, processing, retailing, catering, and preparation, survive typical catering refrigeration temperatures, and increase in number under conditions of thermal abuse [70]. Antibiotic-resistant salmonella infections of both humans and animals are universal concerns, particularly in developing countries, where the risk of infection is high because of unhygienic living conditions, close contact and sharing of houses between animals and humans, and the traditions of consumption of raw or undercooked animal-origin food items [12].

Studies conducted in different parts of Ethiopia have confirmed the presence of *Salmonella* in human beings, different food animals, and food of animal origin. Despite there are reports on prevalence and distribution of *Salmonella* species in the country, the problem of these pathogens in food of animal origin is still not well known. Moreover, the risk factors associated with the contamination of animal products are not described, and incidence of food-borne salmonellosis is still unknown in the country [12, 70]. Occurrence of carrier food animals, illegal slaughtering of animals in open fields, unhygienic slaughter practices in the abattoirs, and widespread tradition of consumption of raw meat are potential risk factors of salmonella infection in the country [27]. Few published findings on prevalence of nontyphoidal *Salmonella* in different parts of Ethiopia are given in Table 2.

*(3) Transmission*. Food-borne transmission is recognized as the major cause of salmonella infections [12]. Animal-origin food and their products are the commonest vehicles of *Salmonella* to humans [64]. However, transmission also occurs by ingestion of water, food contaminated with animal faeces, and contaminated food-processing equipment [3]. The nontyphoidal *Salmonella* serovars are predominantly associated with food of animal origin such as milk, eggs, poultry, beef, and pork [69].

Contaminated animal products usually result from infected animals used in food production or from contamination of the carcasses or edible organs. Cross-contamination of carcasses with *Salmonella* can also occur during slaughtering operations [83]. Eggshells and egg contents can be contaminated by this bacterium during egg formation in the hen reproductive system or from the environment including fecal contact [74]. Fecal or intestinal contamination of carcasses is the principal source of human food-borne infections. The exception is when *Salmonella* is directly transmitted into the food product [67]. The recently emerged *S. typhimurium* DT104, a multidrug-resistant definitive type, is mainly transmitted through ingestion of contaminated beef [3].

*(4) Pathogenesis*. Factors such as strain virulence, infectious dose, route of infection, and host susceptibility mediate the pathogenicity of the organism. Virulence factors such as virulence plasmids, toxins, fimbriae, and flagella help in establishing an infection [64]. The complex invasion process is mediated by the product of a number of chromosomal genes, whereas growth within the host cell depends on the presence of virulence plasmids [8]. Microfold (M) cells are the target cells of *Salmonella* pathogenicity. Some of the mechanisms of pathogenesis are bacterial-mediated endocytosis, neutrophil recruitment and migration, epithelial cell cytokine secretion, fluid and electrolyte secretion, and systemic infection [64].

Normal flora protects against colonization and administration of oral antibiotics facilitates establishment of infection [8]. *Salmonella* avoid host defense in the stomach, reach the intestines, and interact with the nonphagocytic cells such as the epithelial cells of the intestinal mucosa. They adhere to the intestinal epithelial cells by fimbriae (adhesive structures) that promote binding and invade epithelial cells to provoke gastroenteritis [64].

Enteric infection is characterized by local damage without septicemia-salmonella infection with microfold cells in Peyer's patches which is facilitated by fimbrial adhesions. This is followed by ruffling of the target cell membrane which results in internalization of the bacteria in membrane-bound vacuoles. The ruffles facilitate uptake of the bacteria in membrane-bound vacuoles or vesicles which often coalesce [8].

*(5) Symptoms*. The incubation period ranges from 12 to 72 hrs [3]. The clinical manifestation of salmonella infection ranges in severity from self-limiting gastroenteritis to septicemia. The severity of disease depends heavily on the host susceptibility and the virulence of the serovar [64].

Symptoms are usually gastrointestinal including nausea, vomiting, abdominal cramps, and watery, greenish, and foul-smelling diarrhea or bloody diarrhea with mucous. Other findings include headache, prostration, fatigue (muscle weakness), and moderate fever [8]. The disease is of self-limiting nature and does not require specific treatments but can result in serious complication in young children, old, and immunocompromised individuals [3]. Reactive arthritis, sickle-cell anemia, and osteomyelitis due to salmonella infection are much more common in the general population [8].

*(6) Detection*. *Salmonella* surveillance and monitoring should be based on reliable and efficient detection methods, which should help improve food safety. Commercially available rapid methods for *Salmonella* detection can be divided into several categories including new selective media, modified or adapted conventional procedures, immunology-based assays, and nucleic acid-based assays [84].

In general, culturing and isolation of bacteria using selective enrichment media such as *Salmonella* Shigella agar, Hektoen enteric agar, or deoxycholate agar and broth for enrichment are usual procedures [8].

Serotyping has wide acceptance as a method to differentiate *Salmonella* strains, and it is an important tool in public health [67]. Immunology-based methods involving antigen-antibody bindings have been widely used for the detection of food-borne pathogens [85]. The immunology-based assays include ELISA, latex agglutination tests, immunodiffusion, and immunochromatography (dipstick). Moreover, direct hybridization (DNA probe) and amplification (PCR) methods are the two major techniques of the molecular assays that are used for the detection of *Salmonella* pathogens [84].

*(7) Prevention and Control*. Instituting biosecurity and biocontainment practices in addition to enhanced food processing method and preparation and storage practices are required to control and prevent food spoilage due to *Salmonella* [8]. Attenuated DNA recombinant live *Salmonella* vaccines combined with comprehensive control strategy in animals, feed, and animal foodstuff will help to reduce salmonellosis [64].

Safe food preparation practices including through cooking, reheating of food, pasteurization (boiling) of milk, adequate refrigeration, and exclusion of pets and other animals from food-handling areas should be carried out [8].

Additional measures to control secondary contamination could be prevention of contamination by cleaning and disinfection, hygiene of personnel, and proper processing [64]. Vulnerable groups are advised to avoid consuming undercooked meat and poultry, raw milk, eggs, and foods that contain raw egg [8].

#### 2.2.3. *Campylobacter*

*Campylobacter* was first reported in 1886 by Theodore Escherich who observed and described nonculturable spiral-shaped bacteria [86]. The genus *Campylobacter* is of great importance in human medicine and food safety, in addition to its veterinary importance [87]. *Campylobacter* species are the leading cause of bacterial-derived food-borne diarrheal disease in humans worldwide [88], resulting mainly from the contamination of food of animal origin [89, 90]. *Campylobacter* can colonize in most of the warm-blooded animals and poultry [91].

The zoonotic nature of campylobacteriosis makes it important from clinical and economic perspectives worldwide [92]. They are responsible for 15% of food-borne illness-related hospitalizations and 6% of food-borne illness-related deaths [8], and an estimated 400 million cases are reported per year due to *Campylobacter* infection [93, 94]. The economic losses due to *Campylobacter* infections are mainly related to treatment costs, loss of productivity for infected people, and costs for controlling the pathogen [92].

*(1) Etiology*. The word Campylobacter is derived from two Greece words “campylos” meaning curved and “baktron” meaning rod [93]. *Campylobacter* is a member of the family Campylobacteriaceae, which also includes the genera *Arcobacter* and the species *Bacteroides ureolyticus* [4]. The genus *Campylobacter* contains small (0.2–0.8 *μ*m × 0.5–5 *μ*m) [86], Gram-negative, curved, or spiral and microaerophilic bacteria [3, 91, 93, 95] with a distinctive “darting” motility, and they are catalase- and oxidase-positive [89, 90]. When two or more bacterial cells are grouped together, they form an “S” or a “V” shape of the gull wing [86].

The genus *Campylobacter* currently comprises 25 species and 8 subspecies [93]. Among these *Campylobacter* species, thermophilic *Campylobacter* species, particularly *C. jejuni* and *C. coli*, are important food-borne pathogens [3]. *C. jejuni* is the major frequently reported *Campylobacter* species (80% to 90%) followed by *C. coli* (5% to 10%) [93].

*(2) Epidemiology*. *Campylobacter* species are the leading cause of human and animal bacterial diarrheal disease worldwide [89], and they are common problems both in developing and industrialized countries of world [91].

They are found ubiquitous in the environment, and many species act as reservoirs or are susceptible, and wild birds are known to be natural hosts [93, 96]. *Campylobacter* outbreaks are sporadic in nature and are not associated with mortality but can result in secondary complications [3].

These organisms are widely distributed in nature and are mainly recognized as zoonotic infections in a multitude of animal reservoirs, particularly avian species, with shedding into the environment [90]. They are known to colonize the alimentary tract of wild and domesticated birds and mammals, including all food-producing animals [97]. *Campylobacter* can be found in the reproductive organs, intestinal tracts, and oral cavity of animals and humans [89].

100% of poultry (including chickens, turkeys, and waterfowl) [8], cattle, sheep, pigs, and other food animals [86, 87, 98], as well as wild animals and birds [93], are reservoirs of *Campylobacter*, and they harbor high levels of strain diversity. Companion animals including cats and dogs also carry *Campylobacter* species [96], and rodents may act as reservoirs [87]. Poultry is generally considered to be the most important reservoir for *Campylobacter* species [3, 94]. However, table eggs are not considered as an important source of the organism [3]. Organisms can be found in bulk milk samples, tissue specimens from beef cattle, and raw ground beef [87].

A large and diverse number of risk factors contribute to the susceptibility of humans to campylobacteriosis. Traveling is the most important risk factor followed by consumption of undercooked chicken, environmental exposure, and direct contact with farm animals [99]. In Ethiopia, few studies are conducted in the central part of the country to estimate the prevalence of *Campylobacter* species in food of animal origin, and some of the published findings are described in Table 3.

*(3) Transmission*. Transmission can occur through direct contact with infected animals or from equipment, water, or during carcass dressing in a slaughter line [100]. The main transmission route of *Campylobacter* to humans is handling, preparation, and consumption of contaminated food, especially of poultry origin [88, 95]. Animal food products are most commonly contaminated by this pathogen during slaughter and carcass dressing [94] or indirect fecal contamination [98, 101].

Infections occur mainly from consumption of contaminated poultry [94], beef, and pork [9] or other animal meat, meat products, raw (unpasteurized) milk, and/or milk products like cheese [3, 89, 90, 93]. Cross-contamination of ready-to-eat food during preparation by food handlers as well as direct contact with animals have also been identified [89]. Around 30% of all cases of infection were caused by the consumption of poultry, 20–30% of cases caused by pathogens from cattle, and a low percentage of pathogenic strains originating from other sources, including game [9].

*(4) Pathogenesis*. The pathogenesis of campylobacteriosis is not fully understood [101]. Though it is not fully understood, several mechanisms are hypothesized which are responsible for pathogenesis of *Campylobacter* infection [95]. Bacterial motility, mucus colonization, epithelial cell invasion, toxin production, attachment, internalization, and translocation play an important role in the disease development associated with *C. jejuni* virulence [8, 95].

The flagellum which has a coded flagellin gene (flaA) enables the bacterium to reach the attachment sites in the intestine. Several putative virulence factors have been identified in *Campylobacter* which contribute to the motility, intestinal adhesion, colonization, toxin production, and invasion. Adhesion of the pathogen to the intestinal epithelium is important for colonization and to increase the secretion of bacterial toxins [101]. The bacteria produce several cytotoxins that contribute to the development of the disease. Furthermore, *C. jejuni* is able to produce superoxide dismutase enzyme which catalyzes the breakdown of superoxide radicals, and it is one of the bacterial major defense mechanisms against oxidative damage [19, 142].

The organism upon consumption multiplies in the intestinal tract and damages the mucosal epithelium, resulting in self-limiting diarrhea and abdominal pain [3]. *C. jejuni* invades both epithelial cells and cells within the lamina propria [8]. The diarrheal disease may be due to the production of a heat-liable toxin [101].

*(5) Symptoms*. The incubation period varies from 3 to 5 days [3]. In humans, campylobacteriosis is characterized by watery and/or bloody diarrhea, abdominal pain, cramps, fever, malaise, and vomiting [4, 95]. This is especially dangerous for young children who are more prone to dehydration and loss of nutrients, such as sodium and protein, as a consequence of the diarrheal illness [95]. The pathogen is also the main causative agent of “traveler diarrhea.” [93] Toxic megacolon, dehydration, and sepsis may occur especially in the young (<1 year of age) and immune-compromised patients [102].

The most important postinfectious complication of *Campylobacter* infection is Guillain–Barre syndrome (GBS) [87], about 2–4 weeks after infection [8], which is characterized by polyneuritis of the peripheral nerves that may lead to either short-term or lengthy paralysis of the limbs which lasts for several weeks [90, 101, 102]. Other complications can include meningitis, urinary tract infections, and short-term reactive arthritis [8].

*(6) Detection*. Identification methods for *Campylobacter* have traditionally involved the use of selective culture media combined with biochemical tests. Recently, PCR has increasingly been applied in the detection and identification of *Campylobacter* [103].

Recently, campylobacteriosis may be diagnosed using conventional and molecular techniques. Bacterial culture is frequently used for routine isolation, and biochemical tests are used for phenotypic identification of *Campylobacter* [92]. Several of the selective broths, e.g., Bolton broth, *Campylobacter* enrichment broth, and Preston broth, have been compared for their efficacy. Several selective agars have been formulated and tested for their efficacy in isolating *Campylobacter*. For example, Preston, charchoal-cefoperazone-deoxycholate, and Butzler agars have been found to be equally effective [86].

As an alternative to growth on agar and biochemical identification methods, a variety of technologies including immunoassay methods and molecular techniques (PCR) can be used [101].

*(7) Control and Prevention*. Control depends on sanitation and hygiene in livestock barns to reduce the bacterial populations in the environment of the animals. The number of organisms can be reduced and controlled in meat-processing plants by using HACCP including washing, handling, and freezing of carcasses. Improvement of food-handling skills in restaurants and in home kitchen will reduce transmission of the organism, and adequate cooking of raw meat such as poultry to an internal temperature of 82°C will eliminate the organism [8].

Biosecurity measures and personal hygiene practices are the main approaches needed to be considered to control campylobacteriosis. Essential oils, prebiotics, probiotics, bacteriocins, bacteriophages, and immunization measures also have a significant role to control *Campylobacter* [99].

#### 2.2.4. *Listeria monocytogenes*

*L. monocytogenes* is a food-borne pathogen widely distributed in nature [104]. It is an important cause of diseases in both animals and humans [105]. It is one of the most virulent pathogens that controlling and monitoring agencies across the globe have been trying to contain [106], which is associated with the highest case fatality rate of 30% approximately, unlike infection with other common food-borne pathogens [107].

It is a major food-borne zoonotic bacterium of serious public health concern [106, 108, 109] following consumption of contaminated food of animal origin [110]. *L. monocytogenes* emergence as a food-borne pathogen dates from 1980, with the occurrence of many outbreaks and sporadic cases of listeriosis associated with the consumption of contaminated food [111].

Moreover, the presence of *L. monocytogenes* in food has important economic consequences, such as the withdrawal of products from the consumer marketplace and a decrease in sales of the incriminated products [112]. Due to ubiquitous nature of *Listeria* species and their unique ability to survive across a broad range of environmental stress including pH, temperature, and salt, they are considered as important food-borne pathogens [113].

*(1) Etiology*. The organisms of genus *Listeria* are psychotropic, Gram-positive, motile, facultative anaerobic, nonspore forming, and rod-shaped bacteria [111, 112, 114, 115]. *L. monocytogenes*, *L. ivanovii*, *L. innocua*, *L. welshimeri*, *L. seeligeri*, *L. grayi*, *L. murrayi*, *L. marthii*, *L. fleischmannii*, and *L. weihenstephanensis* are ten species of genus *Listeria* [116].

Although genus *Listeria* currently comprises 10 species, human cases of listeriosis are caused almost exclusively by the species *L. monocytogenes* [117, 118]. *L. monocytogenes* is the principal pathogen in humans and animals [116]. The bacterium is capable of surviving under refrigeration conditions, low pH, and in high salt concentration [3]. It is a facultative intracellular bacterium that is able to grow in temperatures between 0 and 45°C, that supports pH between 4.4 and 9.4, and that requires minimum water activity of 0.92 [119].

*(2) Epidemiology*. The occurrence of *L. monocytogenes* is worldwide [112]. Listeriosis occurs in a sporadic or epidemic form throughout the world [120].

Genus *Listeria* contains ubiquitous bacteria that are extensively disseminated in normal environments [106, 117, 118]. *Listeria* species are most commonly found in raw food, vegetables contaminated by soil- and water-carrying bacteria, and raw animal products [109, 121].

Even though pasteurization of raw milk destroys *L. monocytogenes*, this process does not eliminate the later risk of contamination of dairy products [115, 122].

*L. monocytogenes* is frequently isolated from food of animal origin such as ready-to-eat meat products, ground beef [111, 115], meat and meat products (sausages) [3, 33], fish and fish products [114], milk, and pasteurized dairy products like soft cheese and ice cream [107, 121].

Human acquisition of listeriosis from animal sources has been shown to occur as an occupational hazard especially in farmers, butchers, poultry workers, and veterinary surgeons [115, 122]. The major risk population groups at risk for invasive listeriosis are the immunocompromised hosts such as pregnant women, unborn or newly delivered infants, organ transplant recipients, cancer and acquired immune deficiency syndrome (AIDS) patients, and the elderly [115].

In Ethiopia, published information occurrence and distribution of *L. monocytogenes* and food-borne listeriosis is very limited both in veterinary and public health sectors [107, 108, 110]. Some of the published information on the prevalence of *L. monocytogenes* in food of animal origin in different regions of Ethiopia is described in Table 4.

*(3) Transmission*. The most common route of infection in humans is consumption of food of animal origin contaminated by *L. monocytogenes* [115, 116, 118, 120, 124, 125]. The risk of infection is higher in pregnant women, newborns, older people, and immunocompromised people [3].

*(4) Pathogenesis*. *Listeria* possesses unique virulence factors to invade host, evade immune cells, and cause infection [120]. *L. monocytogenes* has D-galactose residues on its surface that can attach to D-galactose receptors on the host cell. These host cells are generally M cells and Payer's patches of the intestinal mucosa. Once attached to these cells, *L. monocytogenes* can cross the intestinal membrane and enter to bloodstream. The bacterium becomes a blood-borne (septicemic), and it can grow after it enters into host's monocytes, macrophages, or polymorphonuclear leukocytes [8].

Since it is an intracellular bacterium, it has the capability to infect a wide range of cell types, cross barriers (intestinal, blood-brain, and placental barriers) and cause infections [113].

*(5) Symptoms*. The symptoms of listeriosis usually last 7–10 days [8]. *L. monocytogenes* generally presents itself with typical “food poisoning” symptoms [126]. Infection is characterized by flu-like symptoms such as fever, fatigue, and gastrointestinal symptoms (nausea, vomiting, and diarrhea) [106, 120, 121].

It can cause severe life-threatening infections such as septicemia, meningitis, meningoencephalitis, spontaneous abortion, still birth, or fetal infection in high-risk groups [117, 126, 127]. The disease is characterized by high mortality rate in affected individuals, and deaths due to listeriosis in human population increased in the 21^st^ century [3].

*(6) Detection*. The methods include enrichment in selective media, subsequent plating on agar plates, and various tests for species identification [128]. Enrichment procedures are required for this organism [8]. The two-stage enrichment method for detection of *L. monocytogenes* with isolation on polymyxin acriflavin lithium-chloride Ceftazidime aesculin mannitol (PALCAM) agar and Oxford agar is widely used. The Christie–Atkinson–Munch–Peterson (CAMP) reaction is useful for identifying *Listeria* species. This test uses horse blood agar and streaks off hemolytic *S. aureus* and *Rhodococcus equi* in combination with *Listeria* isolates. As molecular methods are accurate, sensitive, and specific, they are increasingly used in identification of *L. monocytogenes* from food [111].

Among molecular methods, PCR and RT-PCR are now established methods for identification of *L. monocytogenes* from other nonvirulent *Listeria* species from food [104, 111]. Using monoclonal antibodies, ELISA has been developed to identify *Listeria* in food [8].

*(7) Control and Prevention*. Preventing listeriosis can be done by carrying out an effective sanitation of food contact surfaces [8]. Food-safety control measures must be implemented properly to limit listeriosis and to ensure consistent control strategies. Good hygiene, GMP, and sanitation are the most appropriate strategies in operating procedures [120]. Vulnerable individuals (pregnant women, the elderly, and the immune-suppressed) are advised to avoid consuming unpasteurized dairy products to reduce the risk [111].

Standardized legal regulations and control of meat product manufacture should be a fundamental way to protect food from *L. monocytogenes* contamination. One of the most important ways to protect food from these microorganisms is to prevent the spread of the bacteria at processing plants at different stages of the food production chain [129].

The implementation of HACCP approach and the establishment of effective critical control points can significantly reduce the level of *Listeria* contamination in many processed food products. Standards/legislation for the pasteurization of ice cream/frozen desserts adapted in various countries have an importance in reducing listeriosis [111].

#### 2.2.5. *Escherichia coli*

*E. coli* is among many pathogenic microorganisms which can get access to food of animal origin and is considered as a reliable indicator of contamination by manure, soil, and contaminated water [23]. An emerging clonally distinct form of *E. coli* was first identified as a significant food-borne zoonotic pathogen in 1982 when it was associated with an outbreak in the USA of severe bloody diarrhea that was traced to the consumption of undercooked hamburgers [130].

Most of *E. coli* are normal inhabitants of the gastrointestinal tract (lower ileum and large intestine) of animals and humans [23, 131, 132], while others are pathogenic to humans [133]. *E. coli* are of zoonotic in nature and constitute a public health hazard [134]. Shiga toxin-producing *E. coli* were associated with several life-threatening food-borne outbreaks worldwide [37].

*(1) Etiology*. *E. coli* is characterized as a Gram-negative, rod-shaped bacterium belonging to the family Enterobacteriaceae with five virulence groups, including enteroaggregative *E. coli*, enterohemorrhagic *E. coli*, enteroinvasive *E. coli*, enteropathogenic *E. coli*, and enterotoxigenic *E. coli* [27]. They are rod-shaped bacteria up to 3 *μ*m in length and can ferment glucose and other sugars. They are motile with peritrichous flagella and often fimbriated [8].

*E. coli* O157:H7 is one of the best known serotypes to contain pathotypes that can cause food-borne infection in humans [17, 27, 35, 133]. It is a well-known Shiga toxin-producing bacterium [29, 135], and it is a major food-borne and zoonotic pathogen [132].

*(2) Epidemiology*. *E. coli* O157:H7 is one of the most important food-borne pathogens [136, 137]. *E. coli* O157:H7 has been reported increasingly from all parts of the world [17]. This strain is an important emerging food-borne pathogen of humans causing outbreaks worldwide [3, 138]. Outbreaks have been reported from different countries of the world including the United States, Canada, Asia, Australia, Europe [23], and different African countries, stretching from South to East and West parts of the continent [131]. It has been indicated that an estimated 74,000 cases and 61 deaths annually are attributable to *E. coli* O157:H7 in the USA [17].

*E. coli* is a normal part of the intestinal microflora of many healthy animals, including humans. However, some strains can cause diseases [136]. Cattle are the major reservoirs of *E. coli* O157:H7 [3, 8, 131, 133, 139] followed by sheep and goats [137]. The role of small ruminants as a source of human infection through fecal shedding is being reported in a number of studies [131]. The organisms are also isolated from horses, dogs, and deer [3]. Milk and milk products [134], poorly cooked beef, tainted ground beef, and other bovine food products are identified as major sources of infection in outbreaks [139, 140].

The rising incidence and potentially serious nature of *E. coli* O157:H7 infection are a cause for concern to public health authorities [131]. The major contributing factors of *E. coli* O157:H7 infection are changes in eating habits, mass catering, complex and lengthy food-supply procedures with increased international movement, and poor hygiene practices [29].

Epidemiology of food-borne pathogens especially that of *E. coli* O157:H7 was not well studied in Ethiopia in the past years [135]. According to Abreham et al. [131], the data on the prevalence, virulence, and gene diversity in ruminants and food of animal origin in Ethiopia are still limited. However, recently, there is an increasing trend of reporting occurrence level of the organism in beef and dairy products [135]. Though most of the studies were conducted in central Ethiopia, studies have been carried out to estimate the occurrence of *E. coli* in food of animal origin, mainly in meat and milk in recent years. Some of the published findings are described in Table 5.

*(3) Transmission*. The most frequent mode of transmission for *E. coli* O157:H7 infection to humans is through consumption of contaminated food and water and may also spread directly from person to person and occasionally through occupational exposure [133]. *E. coli* are food-borne zoonosis transmitted through the consumption of contaminated food of animal origin [134]. The most common means of transmission of *E. coli* O157:H7 to humans is through consumption of raw or undercooked meat of bovine origin [131, 133, 136, 137]. Transmission may occur through consumption of contaminated raw milk [3, 35] and unpasteurized dairy products [132]. Nevertheless, varieties of other food items have also been implicated in causing outbreaks [131].

Carcass contamination occurs through skin-to-carcass or fecal-to-carcass transfer of the pathogen during slaughter process at processing plants which is the major risk factor for human infection. Furthermore, cross-contamination can occur during further processing of carcasses in the processing plants, during distribution and storage of beef at retail markets [139].

*(4) Pathogenesis*. Virulence factors, Shiga-like toxin, and adherence factors are the mechanisms of bacterial pathogenicity [142]. Intimin gene is responsible for the bacteria's intimate adhesion to intestinal cells, causing the appearance of attachment lesions and erasure of the microvilli of the brush border of enterocytes [131]. *E. coli* consists of the destruction of microvilli, intimate effacing adherence of the organism to the enterocyte membrane, and changes in the cytoskeletal structure of the enterocyte associated with the accumulation of polymerized actin and other cytoskeletal proteins beneath the site of bacterial attachment [143].

The adhesive structures may be fimbrial or nonfimbrial, which would mediate the process of binding and colonization. Adhesive factors may lead to bacterial colonization, proliferation, and pathogenesis. *E. coli* O157:H7 can adhere to intestinal mucosal tissues and secrete several proteins, enzymes, and toxins [142]. The production of Shiga toxin is central to the pathogenesis of bloody diarrhea and haemolytic uraemic syndrome. *E. coli* O157:H7 strain produces Shiga toxin 1 (stx-1) and Shiga toxin 2 (stx-2) [138].

The toxins provoke cell secretion and kill colonic epithelial cells. In animals, the toxin has been shown to cause localized fluid accumulation and colonic lesion characterized by sloughing of the surface and crypt epithelial cells [8].

*(5) Symptoms*. The incubation period ranges between 2 and 10 days followed by appearance of diarrhea, abdominal pain, vomiting, hemorrhagic colitis, haemolytic uraemic syndrome with acute kidney failure, and thrombotic thrombocytopenic purpura [3, 137]. The clinical sign initially may be diarrhea with abdominal cramps, which may turn into grossly bloody diarrhea in a few days. However, there is no fever. Septicemia starts with bacteremia and ends with toxemia, and its severity depends on the effect of bacteria localization in a variety of tissue spaces throughout the body [8].

*(6) Detection*. During outbreak investigations, surveillance, and quality control, employing sensitive methods to detect *E. coli* O157:H7 is suggested [131]. Different methods such as culture in special media, serological, and molecular assays have been used for detection of this serotype in food, environmental, and clinical samples [144].

Culture isolation of *E. coli* O157:H7 still is the gold standard for identification [142] assisted by biochemical tests [145]. Sorbitol-MacConkey agar (SMAC) supplemented with cefixime and potassium tellurite is one of the most sensitive and differential media to isolate *E. coli* O157:H7 from other serotypes of *E. coli* [143, 144]. The SMAC agar consists of bile salts, a carbohydrate source, sorbitol, and an indicator. *E. coli* O157:H7 does not ferment sorbitol. If *E. coli* O157:H7 is present, colourless colonies will appear, while other Enterobacteriaceae will show up as pink colonies [145]. Their identity is confirmed by agglutination with specific antiserum [146].

Various immunoassay techniques [8], several rapid screening methods, and serological techniques may be helpful for specific diagnosis [143]. PCR-ELISA method, several qPCR methods, loop-mediated isothermal amplification (LAMP), and a magnetic microparticle are the main effective methods to detect Shiga toxins as reported by different scholars [142].

*(7) Control and Prevention*. The prevention/avoidance of food-borne illness caused by *E. coli* can be prevented by the same method as prevention of other food-borne diseases caused by bacteria. However, special precaution is required due to their severe consequences in young children [8].

Preharvest control of *E. coli* O157:H7 is a control strategy which improves human health, food and water safety, and cattle pathogen-reduction by good sanitation procedures during food preparation, practices, and transport management [142]. Another option in cattle is intervention methods such as probiotics, vaccination, antimicrobials, sodium chlorate, and bacteriophages which are important to increase herd resistance to infection and subsequent control of *E. coli* O157:H7 [143]. Vaccination is an attractive strategy to reduce *E. coli* O157:H7 colonization [142].

Postharvest intervention technologies such as skin and carcass washing and the use of antimicrobials have been developed with varying success [139]. Food safety in the processing sector is based on HACCP approach. This approach is not specific to the control of *E. coli* but broadly addresses biological, chemical, and physical hazards. HACCP programs in the processing sector include microbial testing of the product for indicator bacteria and/or specifically for *E. coli* O157:H7. Interventions include the use of chemicals or antimicrobial products, knife trimming, vacuuming, washing, stream pasteurization, multiple hurdle interventions, gamma irradiation, low-dose low-penetrating radiation, and good manufacturing practices in the processing line. A systematic review of retail and consumer food-safety programs found evidence that food-handler training, food premise inspections, and community-based education programmes promoting proper food handling and preparation techniques are effective components in reducing public exposure to food-borne pathogens [143].

## 3. Conclusion and Recommendations

Food-borne zoonotic bacterial pathogens are the leading causes of human illness worldwide with a great burden in developing countries resulting in huge economic loss in addition to public health issues. *S. aureus*, *Salmonella* species, *Campylobacter* species, *L. monocytogenes*, and *E. coli* are the common bacterial pathogens which are associated with food of animal origin. These bacteria may enter into the food chain from production of food animals up to the final consumption of animal products. Currently, these bacterial pathogens have a great concern for public health due to the emergence of multidrug-resistant strains. Studies conducted in different parts of Ethiopia indicated that these zoonotic bacteria are commonly isolated from food of animal origin particularly in meat and dairy products. Despite there are reports on prevalence of food-borne zoonotic bacteria in food animals, food of animal origin, and humans, the burden of these pathogens in food of animal origin is still not well known, the associated risk factors are not clearly identified, and the incidence of human infections from zoonotic food-borne bacteria is not well documented as a majority of them are identified by conventional methods. Risk factors like the habit of raw animal product consumption, unstandardized slaughtering process, and nonhygienic food-preparation procedures and handling may prone people for food-borne zoonotic bacterial pathogens in the country.

Based on the above conclusion, the following recommendations are forwarded:

Coordinated surveillance and monitoring system for food-borne zoonotic bacterial pathogens should be done to design appropriate and effective control and prevention strategies against these pathogens in developing countries including Ethiopia
The epidemiological information regarding to risk factors and incidence of human infections associated with food-borne zoonotic bacterial pathogens should be established and well documented at national level
  - Good manufacturing practices, standardized slaughtering and pasteurization procedures, and hygienic animal product-preparation techniques should be properly implemented
  - Awareness to the public should be created associated with risk factors of food-borne zoonotic bacterial pathogens based on findings of different researchers
  - These bacterial pathogens should be characterized at molecular level, and possible prevention and control strategies should be developed

## Figures and Tables

**Table 1:** Prevalence of *S. aureus* in food of animal origin in different parts of Ethiopia.

Sample type	No. of examined	No. of positive (prevalence)	Source
Raw bulk milk	168	79 (47)	Enquebaher et al.[51]; Tigray Region, Northern Ethiopia
Naturally soured/fermented raw milk	51	13 (25.4)	
Butter milk	44	14 (31.8)	
Butter	32	8 (25)	
Ethiopian cottage cheese	7	2 (28.6)	
Cheese	4	2 (50)	
Cakes made from milk	4	2 (50)	
**Total**	**310**	**120 (38.7)**	

Abattoir	384	36 (9.4)	Adugna et al. [54]; Addis Ababa city
Butcher	384	76 (19.8)	
Cutting table	40	6 (15)	
Hook	40	6 (15)	
Knife	40	9 (22.5)	
**Total**	**888**	**133 (15)**	

Raw cow milk	170	48 (28.2)	Tessema and Tsegaye [39]; Alage Atvet College Dairy Farm, Ethiopia
Raw cow milk	140	45 (32.14)	Tessema [55]; Wolayta Sodo
Milk shop	86	20 (23.26)	Abraha et al. [43]; Mekelle town
Dairy milk	86	23 (26.74)	
**Total**	**172**	**43 (25)**	

Milk and milk products	291	68 (23.4)	Ayele et al. [56]; Sebeta
Milk	183	28 (15.3)	Regasa et al. [57]; Mukaturi and Sululta town, Oromia Region
Milk	160	78 (48.75)	Daka et al. [58]; Hawassa area
Beef carcass swab	400	137 (34.3)	Hassan et al. [59]; Asella
Raw camel milk	384	44 (11.45)	Serda et al. [60]; Jigjiga district

**Table 2:** Prevalence of zoonotic nontyphoidal *Salmonella* in different regions of Ethiopia.

Sample type	No. of examined	No. of positive (prevalence)	Source
Mutton	200	3 (1.5)	Kassaye et al. [71]; Addis Ababa Abattoirs Enterprise
Chevon	184	1 (0.54)	
Total	384	4 (1.04)	
Cheese	96	3 (3.1)	Liyuwork et al. [75]; Addis Ababa
Yogurt	96	0 (0)	
Butter	96	1 (1.04)	
Milk	96	2 (2.1)	
**Total**	**384**	**6 (1.6)**	

Eggshell	166	4 (2.4)	Taddese et al. [74]; Jimma town
Egg content	166	**5 (**3.01)	
**Total**	**332**	**9 (2.7)**	

Chicken carcass and giblets	378	80 (21.1)	Molla et al. [76]; Debre Zeyit and Addis Ababa
Minced beef	160	23 (14.4)	Ejeta et al. [77]; Addis Ababa
Mutton	85	12 (14.1)	
Pork	55	9 (16.4)	
**Total**	**300**	**44 (14.7)**	

Raw beef	448	56 (12.5)	Wabeto et al. [78]; Wolaita Sodo
Raw cow milk	384	36 (9.35)	Mulaw [79]; Bahir Dar town
Cattle carcass	96	7 (7.29)	Atsbha et al. [80]; Mekelle city
Cattle carcass	300	23 (7.6)	Muluneh and Kibret [81]; Bahir Dar town
Raw cow milk	112	15 (13.4)	Abunna et al. [82]; Modjo town

**Table 3:** Prevalence of *Campylobacter* species in food of animal origin in different parts of Ethiopia.

Sample type	No. of examined	Number of positive (prevalence)			Source
		*C. jejuni*	*C. coli*	Total	
Sheep carcass	218	17 (7)	6 (2.8)	23 (10.6)	Woldemariam et al. [87]; Debre Zeyit area, Ethiopia
Goat carcass	180	12 (6.7)	5 (2.8)	17 (9.4)	
**Total**	**398**	**29 (7.3)**	**11 (2.76)**	**40 (10)**	

Beef	227	12 (5.3)	2 (0.9)	14 (6.2)	Dadi and Asrat [89]; Addis Ababa and Debre Zeyit
Mutton	114	10 (8.8)	2 (1.7)	12 (10.5)	
Chevon	92	5 (5.4)	2 (2.2)	7 (7.6)	
Pork	47	1 (2.1)	2 (4.3)	3 (6.4)	
Chicken	60	11 (18.3)	1 (1.7)	12 (20)	
**Total**	**540**	**39 (7.2)**	**9 (1.7)**	**48 (8.9)**	

Beef	384	28 (7.3)	8 (2.1)	36 (9.4)	Faris [28]; Addis Ababa
Carcass swab (mutton)	70	14 (20)	1 (1.4)	15 (21.4)	Chanyalew et al. [100]; Debre Birhan

**Table 4:** Prevalence of *L. monocytogenes* in food of animal origin in different areas of Ethiopia.

Sample type	No. of examined	No. of positive (prevalence)	Source
Mutton swab	768	29 (3.8)	Mulu and Pal [107]; Addis Ababa
Cutting table	45	4 (8.9)	
Hook	20	0 (0)	
Knife	40	3 (7.5)	
**Total**	**873**	**36 (4.1)**	

Raw meat	60	4 (6.6)	Garedew et al. [110]; Gondar town
Minced beef	25	3 (12)	
Fish meat	50	3 (6)	
Pizza	24	2 (8.3)	
Pasteurized milk	50	0 (0)	
Raw milk	50	2 (4)	
Cottage cheese	40	0 (0)	
Ice cream	20	3 (15)	
Cream cakes	65	7 (10.7)	
**Total**	**384**	**24 (6.25)**	

Raw meat	—	4/59 (6.8)	Derra et al. [105]; area of Addis Ababa
Raw milk	—	2/59 (3.4)	
Cottage cheese	—	1/59 (1.7)	
Cream cake	—	3/59 (5.1)	
**Total**	**240**	**10 (4.1)**	

Cottage cheese	100	1 (1)	Gebretsadik et al. [123]; Addis Ababa
Raw beef	76	2 (2.6)	
Raw milk	100	13 (13)	
Liquid whole egg	115	5 (4.3)	
**Total**	**391**	**21 (5.4)**	

Minced beef	61	1 (1.63)	Molla et al. [108]; Addis Ababa
Chicken	52	1 (1.9)	
Fish	43	1 (2.3)	
Pork	53	4 (7.5)	
Cottage cheese	61	0 (0)	
Ice cream	46	9 (19.6)	
**Total**	**316**	**16 (5.1)**	

Raw milk	343	7 (2.04)	Seyoum et al. [122]; Central Highlands of Ethiopia
Pasteurized milk	65	13 (20)	
Yogurt	20	1 (5)	
Cheese	15	4 (26.7)	
**Total**	**443**	**25 (5.6)**	

**Table 5:** Prevalence of *E. coli* in food of animal origin in different areas of Ethiopia.

Sample type	No. of examined	Number of positive (prevalence)		Source
		*E. coli*	*E. coli* O157:H7	
Beef	113	35 (30.97)	3 (2.65)	Taye et al. [132]; Harumaya
Beef	128	—	17 (13.3)	Bekele et al. [133]; Addis Ababa
Mutton	128	-	12 (9.4)	
Chevon	128		10 (7.8)	
**Total**	**384**	-	**39 (10.2%)**	

Raw cow milk	380	129 (33.9)	11 (2.9)	Disassa et al. [23]; Asosa town
Cheese	35	14 (40)	2 (5.71)	Bedasa et al. [17]; Bishoftu town, Central Ethiopia
Raw milk	25	8 (32)	3 (12)	
Pasteurized milk	40	0 (0)	0 (0)	
Yogurt	35	9 (25.71)	0 (0)	
Meat	65	9 (13.84)	2 (3.07)	
**Total**	**200**	**40 (20)**	**7 (3.5)**	

Beef	250	—	20 (8)	Hiko et al. [136]; Debre Zeyit and Modjo
Mutton	243	—	6 (2.5)	
Chevon	245	—	5 (2)	
**Total**	**738**	—	**31 (4.2)**	

Carcass swab	150	—	14 (9.33)	Haile et al. [29]; Jimma town
Carcass swab	110	—	5 (4.5)	Beyi et al. [141]; central Ethiopia
Cutting board swab	110	—	4 (3.6)	
Fecal sample	370	—	7 (1.89)	Abdissa et al. [139]; Addis Ababa and Debre Birhan cities
Skin swab	370	—	2 (0.54)	
Intestinal mucosal swab	370	—	3 (0.81)	
Carcass internal swab	370	—	2 (0.54)	
Carcass external swab	370	—	0 (0)	
Environmental swabs	62	—	0 (0)	
Cutting board	125	—	1 (0.8)	
